# Sustainable Reuse of Waste Tire Textile Fibers (WTTF) as Reinforcements

**DOI:** 10.3390/polym14193933

**Published:** 2022-09-20

**Authors:** Ali Fazli, Denis Rodrigue

**Affiliations:** Department of Chemical Engineering, Université Laval, Quebec, QC G1V 0A6, Canada

**Keywords:** waste tire textile fibers, soil composite, reinforced concrete, asphalt mixtures, rubber aerogels, polymer blend

## Abstract

Waste tire textile fibers (WTTF), as a by-product (10–15% by weight of tires) of end-of-life tires (ELT) mechanical recycling (grinding), are classified as hazardous wastes and traditionally burnt (thermal recycling) or buried (landfilling), leading to several environmental and ecological issues. Thus, WTTF still represent an important challenge in today’s material recycling streams. It is vital to provide practical and economical solutions to convert WTTF into a source of inexpensive and valuable raw materials. In recent years, tire textile fibers have attracted significant attention to be used as a promising substitute to the commonly used natural/synthetic reinforcement fibers in geotechnical engineering applications, construction/civil structures, insulation materials, and polymer composites. However, the results available in the literature are limited, and practical aspects such as fiber contamination (~65% rubber particles) remain unsolved, limiting WTTF as an inexpensive reinforcement. This study provides a comprehensive review on WTTF treatments to separate rubber and impurities and discusses potential applications in expansive soils, cement and concrete, asphalt mixtures, rubber aerogels and polymer composites.

## 1. Introduction

As a consequence of a significant increase in the number of vehicles, more than 1.2 billion tires are manufactured annually, generating a large quantity of scrap tires ending up in landfills over the years and causing significant damage to the environment [1]. The management of used tires from its inception to its final disposal has become a major concern around the globe [2,3]. It is of great importance to develop safe and efficient disposal techniques for these waste streams, which has been an important challenge to the environment and ecosystems [4,5]. Based on a report of the United States Tire Manufacturers Association (USTMA), about 4.2 million tons (249 million scrap tires) were generated in the US in 2017, while the Canadian Association of Tire Recycling Agencies (CATRA) reported the generation of about 386 thousand tons of scrap tires in 2016 [6,7]. Similarly, data from the European Tire and Rubber Manufacturers Association (ETRA) showed that 3.6 million tons of scrap tires were generated in Europe in 2013, while 51 million waste tires were discarded in Australia between 2013 and 2014 [8,9]. All these numbers point towards the generation of high amounts of waste tires to be managed. Table 1 presents a brief overview of a tire composition in which each component in a tire formulation contributes to a specific characteristic, leading to a long lifetime, and achieves dimensional stability with high performances under severe conditions (high temperatures, radiations, mechanical stresses and chemical reagents) [10,11].

Vulcanized tire rubber as an elastic, insoluble and infusible thermoset material containing about 1.52–1.64% sulfur requires several decades to degrade, naturally causing significant health and environmental problems if not recycled or discarded properly [12,13]. The main applications for end-of-life tires (ELT) disposal include reusing (5–23%), landfilling (20–30%), energy recovery and pyrolysis (25–60%), as well as blending with other materials to produce composites (3–15%) [14].

**Figure 1:**
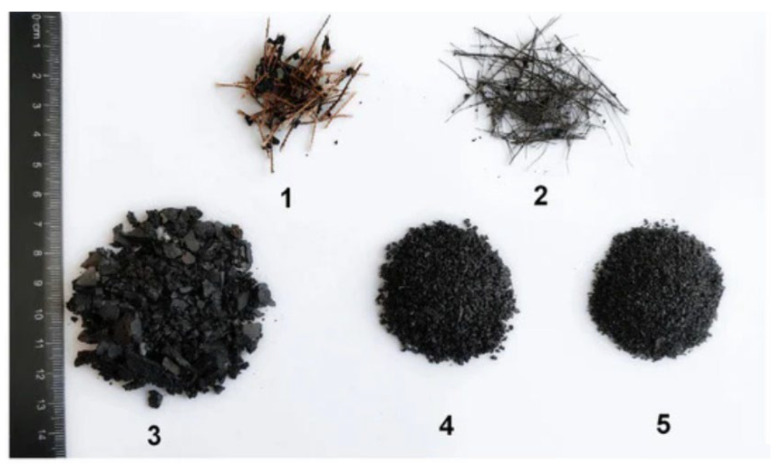
The different materials extracted from end-of-life tires such as (1) textile fiber, (2) metal cord, (3) large size fraction of rubber crumb (1–5 mm), (4) mean size fraction (1–2 mm), and (5) small size fraction (0.1–1 mm) [15].

Figure 1 shows that ELT can be processed into rubber granulates, such as rubber chips, rubber crumbs and rubber powder, as well as other components from tire reinforcement: steel and textile fibers [15]. Besides rubber as the main component of used tires, steel wires and textile fibers are two by-products derived from the treatment of ELT, but both are generally discarded as undervalued resources. The separated steel wires can simply be used in tunnel linings, hydraulic structures, bridge decks, pavements and slope stabilization [16]. Furthermore, waste tire textile fibers (WTTF) are generated in significant amounts (10–15% by weight of waste tires) and are classified as special wastes (EWC code 19.12.08) which have to be carefully disposed of or incinerated [10].

**Figure 2:**
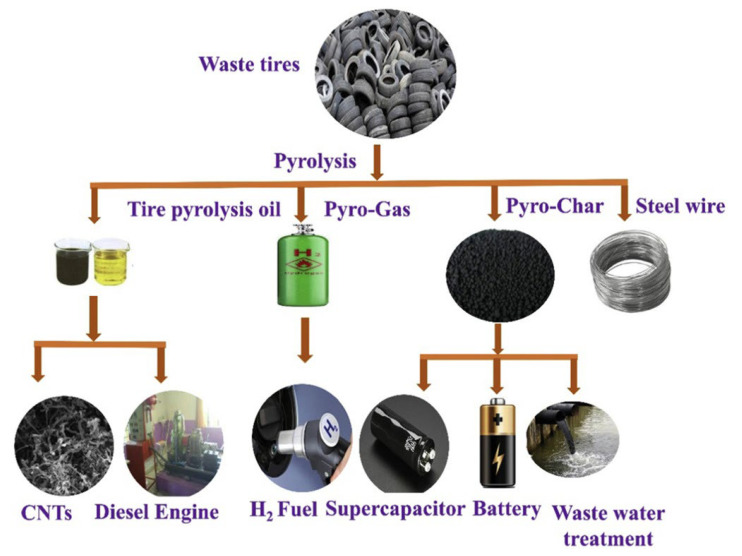
Classification of the different waste tire pyrolysis products with possible applications [21].

ELT can be reused by stripping off the tire tread and applying a new one via cold or hot processes, which is called retreading with the purpose of increasing the lifetime of used tires. Although tire retreading is an ecofriendly and waste-free method of ELT disposal, the low quality and safety concerns at high speeds restrain this approach for passenger car tires [2,13]. Burying ELT in landfills causes important environmental issues since not only the materials occupy large spaces, but they are the origin of self-initiated fires during hot seasons, as well as storing rain water, creating a breeding habitat for rodents and insects [17,18]. About 49% of waste tires are incinerated to produce energy since tire rubber benefits from a high calorific value (32.6 MJ/kg), competing with common fuel to produce steam, electrical energy, pulp, paper, lime and steel [2]. However, because of incomplete/inefficient combustion, tire combustion leads to the emission of air pollutants, such as carbon oxides (CO_x_), nitrogen oxides (NO_x_), sulfur oxides (SO_x_) and polycyclic aromatic hydrocarbons (PAH) [19]. Accordingly, energy generation through tire incineration was banned in most developed countries following the Paris agreement to prevent air pollution. However, pyrolysis can be considered as a practical strategy to transform ELT as an available source of hydrocarbons into gases, carbon blacks, liquid fuels, pyrolytic oils and pyrolysis char through catalytic or non-catalytic reactions above 400 °C in an oxygen-free environment [12,20]. Figure 2 presents a wide range of possible value-added products from the pyrolysis of ELT [21]. However, the main limitations to use pyrolysis at an industrial scale are high costs (purchase, installation and operation), pyrolysis conditions (high temperature with low pressure), and the generation of toxic hydrogen sulfide (gas) as the main by-product [22].

To avoid high environmental pollution associated with ELT landfilling and combustion, it is imperative to develop alternative modes of waste tires disposal and value-added potential applications. Composites production involves turning tire residues into valuable raw materials through compounding (mixing) with different components. As an upcycling strategy, scrap tires are valorized at the end of their service life to develop low costs and sustainable composites [3,11,23]. For instance, tire rubber crumbs have been used as additives or aggregates in automotive parts [24], concretes [25], asphalt mixtures [26,27], pavements [28,29], soil structures [30], etc.

**Figure 3:**
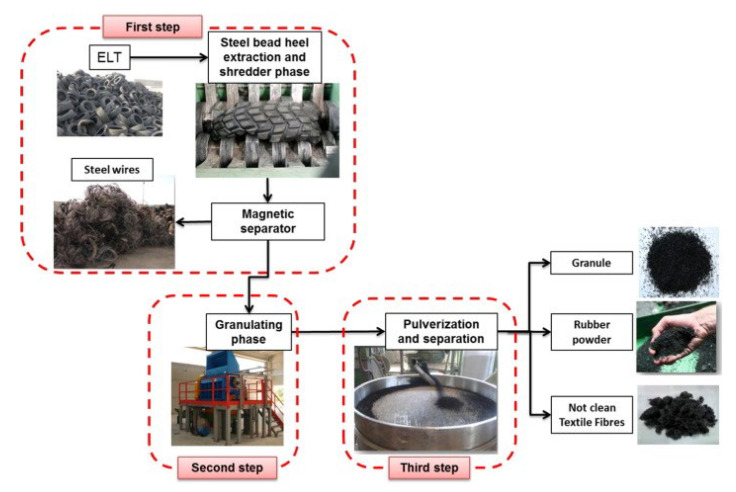
Different ELT disposal processes [10].

Different grinding processes have been developed such as cryogenic, ambient, wet and water jet, converting used tires into triturated rubber particles of various sizes and shapes. The rubber granulates, as the main portion of ELT, can be exploited as aggregates in composite manufacturing with positive effects in terms of cost, weight, mechanical performance, durability and environmental sustainability [31,32]. Figure 3 shows that ELT grinding consists of three main steps. Initially, ground tire rubber (GTR) is produced using grinder blades and knife elements for tire downsizing into 7–10 cm particles followed by the removal of the metallic fraction. Next, crunching is performed to obtain tire rubber granulates of smaller sizes (around 2 cm). Finally, pulverization and separation of the tire residues takes place to produce granulated or pulverized rubber fractions with specific particle sizes (smaller than 1 mm) using pneumatic separators (sieving) and electromagnets for the separation of textile fibers and metal components from the rubbers, respectively. About 15–50% by mass of uncleaned fibers is generated from this process as a mixture of rubber–fiber, which still needs to be fully valorized [10].

Most of the current treatment processes for reusing, recovering, and recycling of ELT are aiming at recovering steel and rubber, while waste management strategies are not implemented for WTTF. This apparent lack of interest is associated with the fact that WTTF represent an obstacle for widespread ELT recycling because of dust accumulating in the workplace, resulting in health problems for operators [33,34]. Moreover, the fibers obtained from the processing of used vehicle tires are contaminated with rubber particles accumulating electrostatic charges, making it difficult for melt processing (inhomogeneous mixing and feeding problems in extrusion or injection), as well as high volume and low specific weight (approximately 140 kg/m^3^), making their transport/handling very difficult and expensive. In addition, rubber particles attached to WTTF contain varying amounts of sulfur, nitrogen, oxygen and hydrogen leading to the emission of toxic gases during combustion [35,36]. As an example, around 5–8 tons of WTTF are buried daily in Iran, indicating the role of tire fiber residues on polluting the soil, underground waters and wildfire, in addition to the high amount of valuable urban lands being filled with this waste [37]. One of the most efficient ways of tackling this challenge is to apply efficient cleaning operations to separate the fine crumb rubber and steel particles from the textile fibers to generate added-value applications [10,38,39].

Recently, alternative strategies for WTTF are gradually being developed based on important environmental and financial incentives instead of landfilling or burning. Among the products acquired in the recycling process of waste tires, WTTF are introduced as promising reinforcements in geotechnical engineering practices [37,40,41], building materials [38,42], and polymer blends [43,44]. According to the literature, Nylon, especially Nylon 6.6, and polyester are the main polymers found in WTTF [10,42,43,45]. WTTF can be incorporated as a reinforcement into different matrices with a processing temperature lower than the melting temperature of Nylon 6.6 (259 °C) [46] and polyester (253 °C) [43] as the main components of WTTF. Conversely, they can be used in “colder” materials such as soil, cement or bituminous conglomerates [10]. For instance, the application of WTTF as reinforcing agents in expansive soil for impermeable liners and covers in landfill applications results in improved geotechnical characteristics such as split tensile strength, swelling–consolidation, volumetric shrinkage and desiccation cracking tests [47].

Since ELT can be of various sources, such as truck tires, off-the-road tires, car tires, tractor tires, etc., the WTTF obtained after grinding have different lengths, diameters and mechanical properties with typical values and characteristics as presented in Table 2. As reported in Table 3, energy dispersive X-ray (EDAX) spectroscopy confirms the predominance of carbon and oxygen in the chemical composition of these fibers, while small amounts of Na, Zn, S, Al and Si are also detected due to the different additives used in vulcanized rubber formulations [40,48].

Some reviews are available on the subject of tire recycling into thermoplastic elastomer (TPE), rubber compounding, building/construction materials, and tire-derived fuels. For instance, the review by Ramarad et al. [2] covers the recycling of the rubber fraction of ELT with a focus on the addition of GTR into polymeric matrices. Fazli and Rodrigue [13] reviewed in detail the curing, rheological, mechanical, aging, thermal, dynamic mechanical and swelling properties of rubber compounds reinforced with recycled tire rubber (rubber review). Bockstal et al. [49] provided a comprehensive review about physical and chemical devulcanization processes used to recycle rubber granules as reclaimed rubber (RR), as well as the properties of rubber compounds filled with RR. Wahid Ferdous et al. [17] reviewed the recent progress using tire rubber residues as replacements of aggregates, binders and fibers in concrete formulations. Compared to a large body of studies on tire rubber waste management, only a few studies were devoted to study the reuse opportunities and potential applications of WTTF in composite preparation and the effect of different fiber treatments on the final product properties. Nevertheless, these treatments are not always very clear and need better understanding to fully exploit the fiber properties into high-value applications.

The current study aims to show that using WTTF in composite materials is an important move towards a circular economy that can minimize virgin materials consumption and develop recyclable fiber-reinforced composites having similar properties to virgin materials at lower costs. In this context, a distinctive effort is made here to summarize all the works dealing with the recycling of treated or untreated WTTF. As a step towards a more sustainable development, a special focus on the possibility of reusing WTTF as reinforcement in soil composites, cementitious materials, asphalt mixture, rubber aerogels and polymer blends is presented and discussed in terms of the advantages and limitations for each application.

## 2. Soil Composite

The creation and expansion of tension cracks and fissures are prevalent phenomena in soil structures such as dams, hydraulic barriers, slopes, runway subgrades, highways and railway embankments [40,50]. In addition, the soil structures used for the construction of impermeable layers (liners and covers in landfills) to prevent from leachate and pollutant migration into the soil and ground water are susceptible to volume changes and desiccation cracking [50]. This is why soil stabilization/reinforcement techniques are being developed to increase the shear and compressive strengths, bearing capacity and permeability of the structures [51]. Chemical techniques are being used for expansive soil reinforcement and stabilization through application of fly ash [52], lime [53], geo-polymers [54], cement [55], and ground granulated blast furnace slag [56]. Furthermore, mechanical techniques have been proposed as a more sustainable solution for expansive soil reinforcement based on randomly distributed synthetic fibers such as polypropylene (PP) [57], polyester [58], polyvinyl alcohol (PVA) [59], waste carpet [60], glass [61], and carbon [62]), as well as natural (biobased) fibers such as kenaf [63], corn husk [64], hemp [65], bagasse [66], sisal [67], and jute [68].

**Figure 4:**
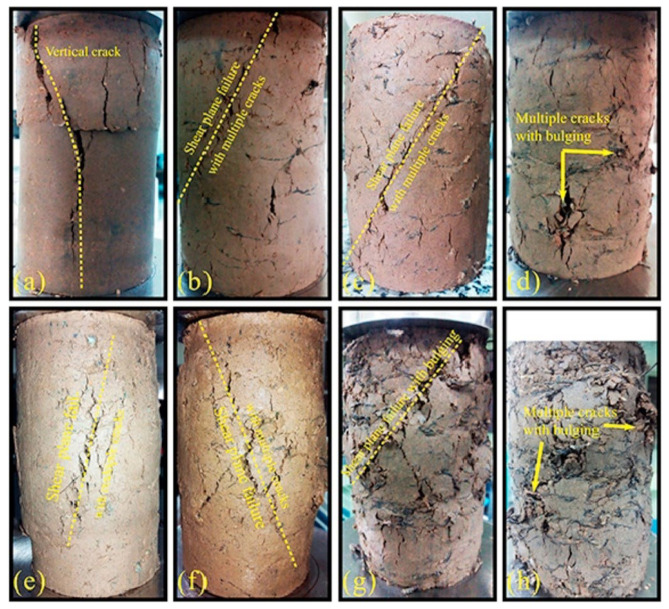
Typical failure patterns under UCS tests: (**a**) CF0, (**b**) CF0.5, (**c**) CF1, (**d**) CF4, (**e**) SF0, (**f**) SF0.5, (**g**) SF3, (**h**) SF4 (clayey and sandy composites are coded as CFχ and SFχ, respectively, where χ represents the weight ratio of WTTF to dry soil) [37].

Tire-derived textile tire fibers are among the main reinforcing agents gaining more attention in geotechnical engineering applications with a special focus on soil structures due to their efficient and economical nature [30,37,41,69]. In one of the earliest attempts towards a new area for WTTF reuse in soil reinforcement, Abbaspour et al. [37] reported that up to 10% was possible to use as an effective soil reinforcement strategy for two types of soils based on clay and sand. As shown in Figure 4, the failure pattern of fiber-reinforced soils in unconfined compressive strength (UCS) tests reveals a brittle failure of the unreinforced specimen due to the presence of a continuous shear plane (vertical crack) from top to bottom (Figure 4a) [37]. Furthermore, incorporation of up to 4% WTTF generated multiple undeveloped cracks and bulging deformation as indicative of the fiber effect to prevent the expansion of the original shear plane leading to higher specimen strength [37].

**Figure 5:**
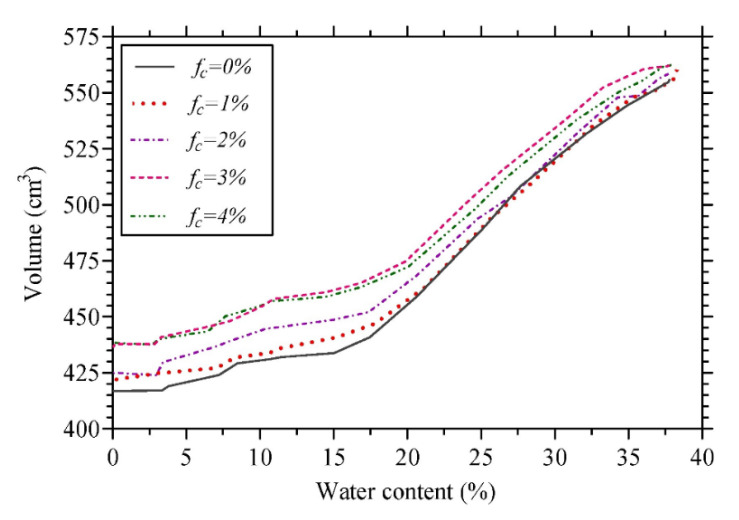
Volumetric shrinkage deformation as a function of water content for different WTTF contents [40].

Expansive soils are mainly used as impermeable liners or covers which are prone to volume changes and suffer from a lack of high load bearing capacity. Therefore, these soils must be modified and improved via various methods prior to construction [40,50]. As a step towards the sustainable development of fiber-reinforced soil structures, Abbaspour et al. [47] and Narani et al. [40] introduced WTTF (2%) as prospective soil reinforcements in sandy soils and expansive clays, resulting in higher load bearing capacity (up to 46.5%), as well as swelling reduction (44%). The overall thickness of the superstructure in flexible pavement/railway structures can be minimized due to improved load-bearing capacity and decreased permanent deformation under repeated loadings of the WTTF-reinforced subgrade soil [70]. Narani et al. [40] studied the effect of WTTF as reinforcing agents on the swelling–consolidation and volumetric shrinkage of the expansive soil. As reported in Figure 5, the volumetric shrinkage strain of the reinforced specimens with different fiber contents (f_c_) of 2%, 3% and 4% decreased by 4.5%, 10.8% and 12.3%, respectively, compared to that of an unreinforced sample in agreement with the results of Olgun [71] who also reported lower volume changes in clay soils stabilized with polypropylene (PP) fiber additions (0.25%, 0.50%, 0.75% and 1.0%). These results confirm that replacing a fraction of expansive solids (soil grains) with WTTF as a non-expansive material with negligible water absorption (highly hydrophobic) leads to lower volume change during drying.

**Figure 6:**
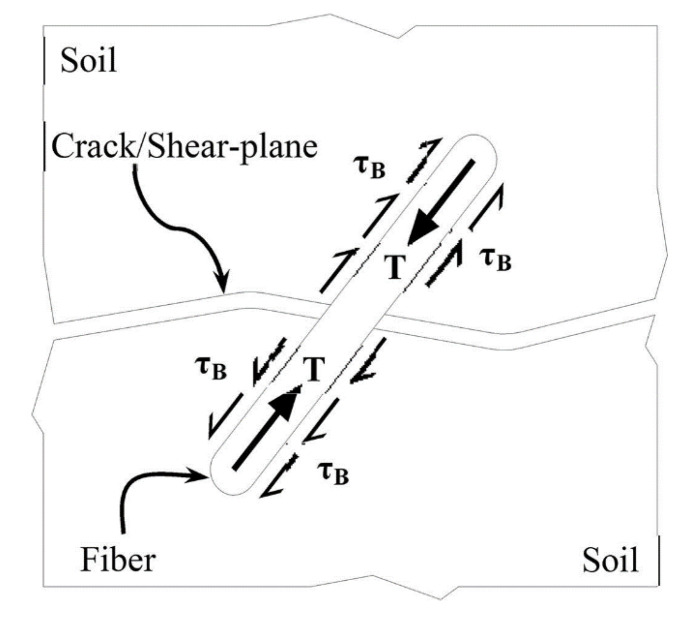
Schematic illustration of fiber–soil interaction (not to scale) [70].

Very few investigations compared the performance/efficiency limitation of WTTF with other fiber options (glass fibers). Valipour et al. [70] investigated the effect of WTTF and glass fibers (0.5, 1.0 and 1.5%) having varying lengths (5 and 10 mm) on the strength/deformation properties of clays. They observed an increasing shear strength of the fiber-reinforced soils in terms of higher internal friction angle (*ϕ_p_*) and cohesion intercept (*c*). The maximum shear strength for the composite soils was obtained at 1.0% glass fibers and 0.5% WTTF because of the reinforcing effects of each fiber and their interaction. As schematically illustrated in Figure 6, after cracking or shear banding/displacement to the fiber-reinforced soil composite, the fibers may be distorted and pulled out from the soil matrix. If the reinforcing fibers can sustain the applied forces before being pulled out from the soil, the stresses within the fibers as a function of the strain level keep the soil element together, leading to increased shear strength. The bonding stress (*τ_B_*) between the fiber and the soil determines the pullout resistance of fibers depending on the adhesion (chemical) and interlocking (mechanical and physical) forces between the fiber and soil, as well as interfacial friction [70,72].

When dealing with different fiber-reinforced composites, it is of great importance to understand how the fibers type, diameter and length can affect the properties and processing of the resulting reinforced samples. For example, an increase in fiber length results in higher probability of fiber agglomeration, which increases the required time of mixing [73]. For example, Tabakouei et al. [73] reinforced sandy soil with three different lengths of fibers including WTTF, date palm (DP) and PP fibers with diameters of 71, 100 and 151 mm, respectively. In WTTF-reinforced soil, contrary to the role of fiber diameter, an increase in fiber length contributed to higher ductility and toughness combined with a drop of the elastic modulus due to the reduced stiffness of longer fibers compared to shorter ones [73].

Very few investigations were carried out on the effect of fiber reinforcement on the performance of subgrade soils. Recently, WTTF-reinforced soil structures were used as subgrade layers of pavements having high function in the distribution of traffic loads [74,75,76]. The incorporation of WTTF into a soil structure may contribute to increasing the resilient modulus (M_R_), which is the material recovery ratio after a series of deviatoric stresses applied to a specimen and expressed as:

(1) $$M_{R}=\frac{\sigma }{\varepsilon_{r}}$$

where σ is the applied stress, and $\varepsilon_{r}$ is the recoverable axial strain. Another possibility is to use the damping ratio, which is the energy dissipated by a vibrating structure caused by dynamic or cyclic loading and is defined as:

(2) $$D=\frac{C}{C_{c}}$$

where C is the damping coefficient, and C_c_ is the critical damping related to the stiffness (k) and the mass (m) via:

(3) $$C_{C}=2\sqrt{\mathrm{km}}$$

In general, the overall performance of such pavements was influenced by interactions between the materials in road layers being complex and rarely studied [76]. Abbaspour et al. [76] investigated the response of soil subgrades as a function of WTTF concentrations by studying the static and dynamic behavior of sandy soil. They also proposed an innovative model for the resilient modulus prediction in fiber-reinforced soils. Suitable interaction and interlocking between WTTF and soil grains was ensured due to simultaneous existence of friction and cohesion to hold the fibers and soil grains together during slippage, leading to increased shear strength. According to their results, adding between 1 and 4% WTTF led to increased shear strength of the fiber-reinforced specimen, reaching up to 17.5% improvement at a 2% fiber content compared to unreinforced soil [76].

## 3. Reinforced Concrete

In recent years, waste tire-filled concretes have been extensively investigated to develop high strength and toughened reinforced concretes. Although the introduction of tire rubber crumbs into cementitious material was reported to improve the resistance to cracking, acid and sulphate attack, chloride ion penetration and sound absorption ability, noticeable decreases in drying shrinkage resistance, as well as compressive and tensile strengths of rubberized concretes were reported. These loses were associated with weak adhesion between rubber particles and the cementitious matrix, as well as the formation of more porous concrete matrices due to rubber particles’ air adhesion/entrapment and hydrophobicity [25,77]. Angelin et al. [78] observed high porosity (void content up to 40%) and significant density reduction (27%) of rubberized mortar due to void spaces entrapped in the cement matrix associated with the presence of rubber aggregates (30%). Contrary to the ductile failure mode with high deformation resistance of rubberized mortar, the compressive and flexural strength of mortars filled with 5% rubber crumbs was reported to be 85% and 96% of the reference specimen, respectively [79]. It is well stablished that the addition of micro- and macro-synthetic fibers changes the brittle behavior of concrete from brittle to plastic or quasi-plastic, resulting in significant improvement of tensile strength, flexural strength, toughness, dynamic load resistance, energy-absorbing capacity and cracking resistance of concrete [80].

**Figure 7:**
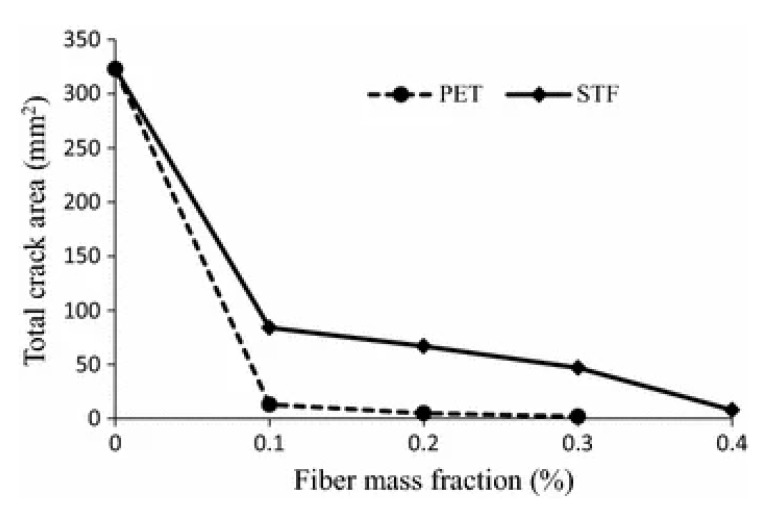
Maximum crack width and total crack area in specimen for STF and virgin PET fibers [42].

Textile fibers derived from ELT can also act as substitutes for polymeric/synthetic fibers such as carbon [81], PVA [82], PP [83], polyethylene (PE) [84], and steel [81] in cement-based materials. These options received increasing attraction to resolve some of the environmental problems caused by waste tires, as well as improving the performance and durability of cement-based materials [38,39,85,86]. Long-term durability and performance of cement composites can be deteriorated by plastic shrinkage cracking caused by self-desiccation and external drying, especially for applications having large surface areas such as slabs on grade, thin surface repairs, patching, tunnel linings, etc. [87]. To overcome this problem, fiber reinforcement of concrete may hinder plastic shrinkage cracking since the fibers can induce bridging forces across the cracks, preventing their growth [88]. Banthia and Onuaguluchi [42] observed that the presence of scrap tire fibers (STF) reduced plastic shrinkage cracking of mortar mixtures with small-sized and segmented multiple cracks compared to large transverse cracks as in reference specimens. They claimed that the presence of tire-derived fibers (0.1–0.4%) and polyethylene terephthalate (PET) fibers (0.1–0.3%) both led to lower maximum crack width of fiber-reinforced mixtures compared to a neat (unreinforced) mortar. Figure 7 shows that lower total crack area was observed for STF and PET fiber-reinforced mortar mixtures by 74–97.5% and 96–99.4%, respectively. This improvement was associated with increased matrix ductility and crack bridging caused by the fibers preventing further crack initiation and propagation, as well as lower capillary pressure in the matrix [42]. Given similar findings in different reports, it was expected to observe higher efficiency of tire fibers in reducing plastic shrinkage cracking compared to that of PET fibers. However, the presence of rubber particles into the tire fibers makes the material fluffier, resulting in an entangled mass of fibers with increased fiber spacing. This state of the material leads to dispersion problems (inhomogeneous distribution) and inaccurate fiber content (fluctuating mixing ratio). All these factors contributed to less effective crack mitigation of the fiber-reinforced specimens compared to specimens containing PET [42]. Similarly, Serdar et al. [89] concluded that compared to neat concrete, the addition of PP fibers and WTTF produced a smaller crack width reflected by the value of restrained shrinkage and higher stresses that the composites can withstand. However, higher WTTF content was required (5 kg/m^3^) compared to PP fiber (1 kg/m^3^) to reach the same properties.

**Figure 8:**
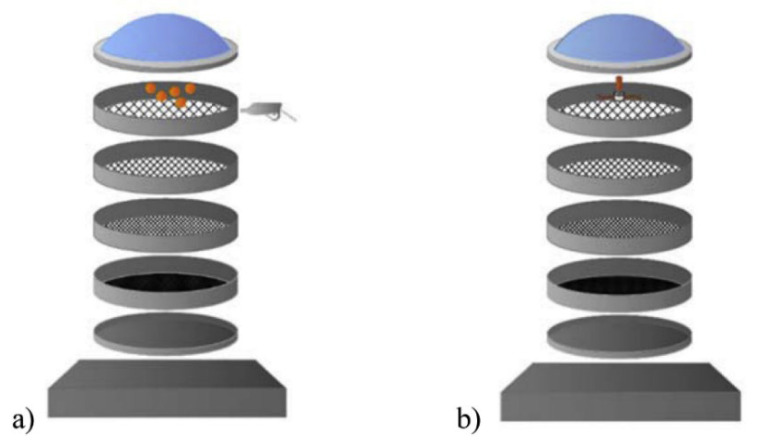
Schematic representation of a fiber cleaning procedure: (**a**) 1st step and (**b**) 2nd step [39].

**Figure 9:**
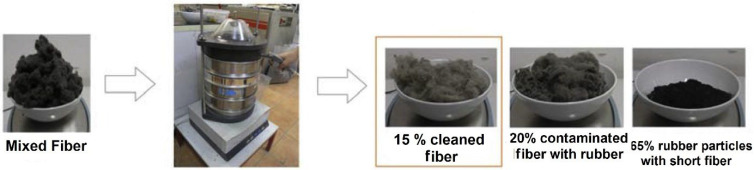
By-products obtained after cleaning mixed fiber [39].

Baričević et al. [39] evaluated the effect of ELT fibers cleaning on the properties of fiber-reinforced concrete. For this purpose, a small-scale cleaning system was designed based on fiber residue shaking and gravitational forces, as rubber particles fell to the bottom of the device. Figure 8 shows that the first step includes a cleaning device consisting of four sieves (openings of 0.25, 0.71, 2 and 4 mm) placed on a shaking table at a frequency of 1.5 Hz, while small rubber balls (top sieve) and compressed air (8 bars) were used to increase the fiber cleaning efficiency. Partially cleaned fibers as a product of the first step were used for further rubber–fiber separation with a shaking frequency and time as in the first step, while a rotating airscrew was placed on top of the cleaning machine (second step) to spin out the contaminated fibers, while all the other impurities fell to the bottom of the sieves. A statistical analysis based on 117 samples showed that the obtained products from the second cleaning step were composed of 65% of rubber particles, 20% contaminated fibers with rubber, and 15% cleaned fibers with respect to the original (as-received) fibers (Figure 9) [39].

Addition of up to 10 kg/m^3^ of uncleaned WTTF and up to 2 kg/m^3^ of cleaned WTTF fibers showed positive effects on the stress distribution and resistance to scaling under freeze–thaw conditions. However, compared to the neat reference mixture, further introduction of cleaned fibers showed up to 5% compressive strength reduction, while higher contamination of uncleaned fibers contributed to more significant decreases (25%) because of higher air content entrapped and residual rubber (crosslinked particles) acting as stress concentration points [39].

To improve on the previous results, several options were proposed by various researchers to optimize the reinforcing efficiency of short fibers used in concrete/mortar through fiber hybridization (two or more types of fibers blended together). The purpose of fiber hybridization is to benefit from synergistic effects generated by each fiber with different lengths or moduli to enhance the overall properties of the resulting composites [81,84].

Chen et al. [90] developed a sustainable cementitious composite reinforced by a combination of recycled tire steel fiber (RTS) (0.5, 1.0 and 1.5 vol%) and recycled tire textile fiber (RTF) (0.5 and 1.0 vol%). To prevent the negative effect of the large numbers of rubber particles attached to the fiber’s surface, weakening the mechanical properties, a sieving method, as proposed by Chen et al. [38], was applied to clean the fibers from the rubber particles and impurities (43.2% of the total mass of as-received fibers). As the compressive strength of fiber-reinforced cementitious composite highly depends on the interfacial interaction between the reinforcing fibers and the matrix, strong fiber–matrix interaction leads to high load-carrying capacity. For example, Chen et al. [90] reported a 76.1% improvement of the 28-day compressive strength by increasing the RTS fiber content from 0.5 to 1.0 vol% for a fixed RTF content (0.5%). The results were associated with good fiber–matrix interactions within the composite [91]. However, increasing the RTF content from 0.5 to 1.0% at fixed RTS (1.0%) decreased the compressive strength by 23.1% due to excessive fiber content increasing the porosity in the interfacial transition zone (ITZ = the weakest part of concrete mixtures) and cementitious matrix leading to lower compressive strength [90].

**Figure 10:**
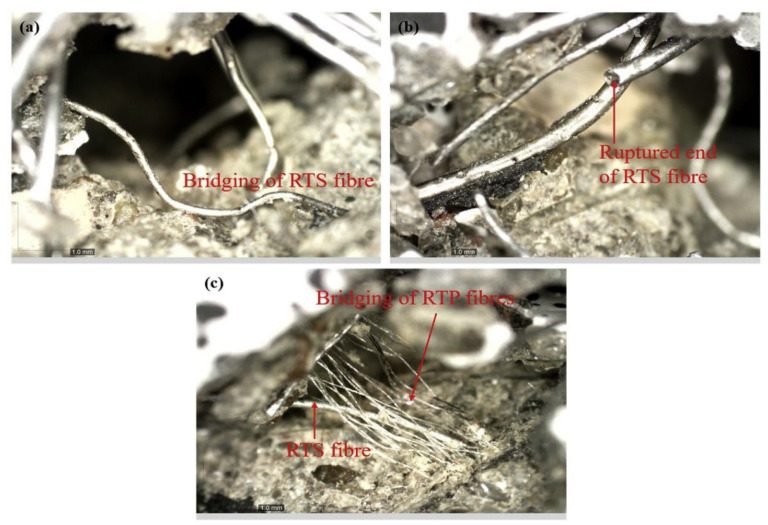
Images of the fiber–matrix interaction taken via optical microscopy [90].

The shear stress caused by the tensile loading transferred to the fiber–matrix interface can be sustained as a consequence of the fiber bridging ability, thus delaying crack growth/propagation [91]. As depicted in Figure 10a, the tensile strength of RTS fibers can resist the shear stress and hence delay the crack initiation and propagation, while the accumulation of tensile stress results in fiber break-up (Figure 10b). However, a hybridization of RTF and RTS (Figure 10c) was more effective to bridge the crack rather than the presence of RTS alone due to a synergistic effect between the combination of these fibers, which not only improved the composites’ strength, but also their post-cracking behavior [90].

In a similar work, Onuaguluchi et al. [36] investigated the use WTTF as a value-added reinforcement in cement composites through hybridization of such microfibers with macrofibers including PP, steel hook-end (HE) and micro/macro scrap tire steel fibers (SSF). Compared to the very low post-crack flexural residual strengths of plain mortar (0.10 MPa), adding WTTF showed negligible improvement (up to 0.20 MPa), while hybridization of 0.35% WTTF with 0.2–0.5% HE and PP fibers led to a noticeable increase in post-crack residual strengths ranging from 1.4 to 3.1 MPa due to the positive synergy between the fibers [36].

Fiber-reinforced concrete with superior crack-resistance and post-cracking performance has a wide range of potential applications including, but not limited to, pavements (airport and highway), bridge decks, tunnel linings and offshore platforms. However, concrete composites are generally subjected to repetitive cyclic loading during their entire service life that may lead to gradual degradation (mechanical fatigue). Thus, optimization of the fatigue behavior of these composites is mandatory [38,92]. Chen et al. [38] investigated for the first time the flexural fatigue behavior of fiber-reinforced concrete using hybridization of WTTF and PP fibers to account for two main factors: stress level and fiber content. They found that for all their reinforced composites, the fatigue life increased with decreasing stress level as reported in similar studies [38]. This behavior can be associated with the high number of initial microcracks and crack propagation induced by cyclic loading at higher stress levels facilitating failure as a result of faster cumulative cracks inside the concrete [38,93]. As expected, the introduction of WTTF not only restricted the formation and development of microcracks due to a fiber bridging effect, but also the rubber crumb attached on the fibers absorbing mechanical energy during fatigue, leading to longer fatigue life [88]. Compared to PP fibers, the distance between WTTF was lower due to their shorter length, increasing the bridging efficiency at a single microcrack within the matrix (more fibers are located inside each individual crack). Thus, WTTF-reinforced concrete showed the longest fatigue life under different failure probabilities [38].

## 4. Asphalt Mixture

**Figure 11:**
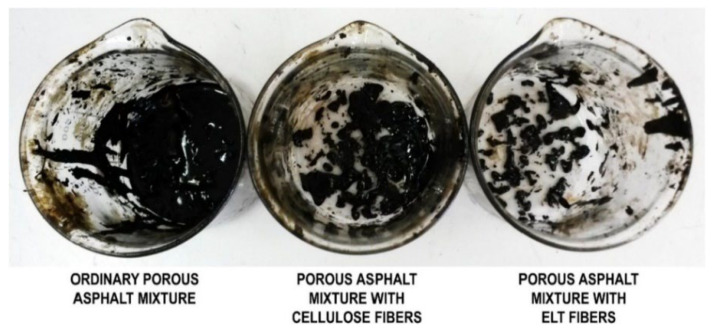
Effect of the fiber type in reducing bitumen drain down from porous asphalt mixtures [99].

The use of ELT to reinforce asphalt mixture is considered as a sustainable solution for paving material to minimize the cost and pollution with favorable engineering benefits [26]. Asphalt mixtures need to be resistant towards permanent deformation and cracking caused by a lack of foundation-bearing capacity and temperature cracking (climatic effects) [94]. For example, fatigue cracking of asphalt pavements is more prevalent in cold climate regions, while asphalt rutting more generally occurs in hot climatic regions [95]. The main advantages of rubberized asphalt mixtures are to delay reflection cracking, reduce traffic noise, minimize maintenance cost, and obtain hardened asphalt mixtures at high service temperatures since tire rubbers do not melt under heat and do not crack at low temperatures [26,28]. Recent studies have identified potential benefits of asphalt mixture reinforcement with recycled rubber derived from ELT grinding processes to produce tire rubber crumbs [27,96]. For instance, it was found that tire rubber crumbs contributed to lower temperature susceptibility and higher resistance towards moisture damage of reinforced asphalt compared with traditional mixtures [4]. Adding tire rubber particles produced better performances in permanent deformation resistance and rutting resistance compared with the unmodified binder and asphalt mixture [97]. Among the various modifiers for asphalt reinforcement, WTTF as a sub-product of ELT treatment have gradually attracted more attention due to their economic and environmental benefits instead of being disposed of in landfills or used in energetic valorization [98]. Waste tire-derived textile fibers have the potential to increase the toughness and fracture resistance of hot mix asphalt (HMA), as well as serve as stabilizers to hinder drain down of the asphalt binder (porous asphalt mixtures and stone mastic asphalt (SMA) mixtures) [94,95,99,100]. WTTF can be considered as a cheap alternative for common fibers such as PP, polyester, asbestos, cellulose, carbon, glass, nylon, coconut, basalt, steel and aramid to lower the use of virgin raw materials and develop asphalt mixtures with good resistance to aging, fatigue cracking, moisture damage, bleeding and reflection cracking [99]. Only few studies investigated the reuse WTTF in asphalt binder modification to induce superior performance of flexible road pavement layers subjected to traffic loading [94,95]. As an early step to introduce textile fibers derived from ELT in asphalt formulations, Putman and Amirkhanian [48] used cellulose, polyester, scrap tire and waste carpet fibers (0.3% wt. for all) to reduce the drain down of SMA mixtures. The results showed that WTTF not only prevented drain down (drain down limit at 10.1%), but also increased the toughness of the mixture without making any significant difference in permanent deformation of fiber-reinforced SMA mixtures. As shown in Figure 11, Landi et al. [99] observed drain down of porous asphalt mixtures composed of a fixed amount of bitumen (5% by mix weight) reinforced by natural (cellulose fibers) and synthetic fibers (tire textile fibers with Nylon 6.6). They placed 500 g of loose mixtures in glass beakers and kept them at 180 °C for 1 h followed by removing the mixtures and weighing the amount of bitumen drained to the bottom of the recipients. The beneficial effect of short fibers to prevent bitumen draining down in porous asphalt mixtures was more significant for WTTF compared to cellulose fibers. This observation might be ascribed to the presence of rubber aggregates stuck on the textile fibers not only reacting with the oily components of bitumen increasing its consistency, but also leading to a thicker bitumen film coating the aggregate particles (better compatibility between the phases present) [99].

**Figure 12:**
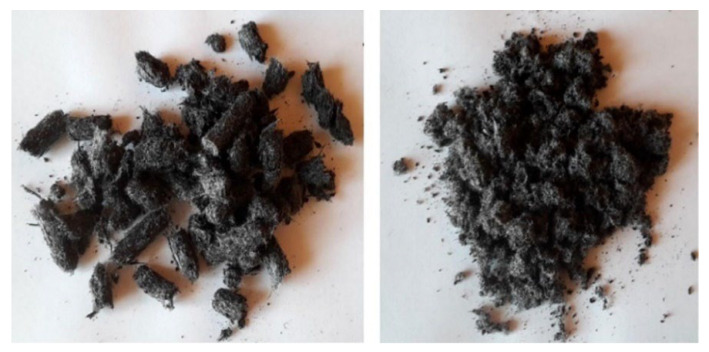
Fiber pellets obtained with (**left**) and without (**right**) paraffin wax [10].

Landi et al. [10] proposed an innovative approach to apply ELT fibers in asphalt focusing on cleaning the fibrous material and its subsequent reuse as a secondary raw material (compacted into pellets) in asphalt mixtures. In particular, they designed a new system for ELT treatment in which the whole discarded tires were transformed into ground rubber particles by grinding (downsizing) followed by the removal of the metallic fraction [10]. The fiber cleaning and compaction process to produce pellets were developed to reuse textile fibers (contaminated with 60% rubber) and facilitated their transport and dosage during the bitumen production [10]. The fiber cleaning step was carried out using a dry washing machine made up of a drum and reel with forward movement through a rotational motion, while fiber compaction was performed below the melting temperature of Nylon 6.6 (259 °C) as the main component of WTTF using another machine by applying a pressure vertically on a grid through the rotation of two cylinders around the main axis [46]. As shown in Figure 12, the use of a paraffin wax was necessary for pellets production to allow for higher fiber compaction (10 times increase in density). The resulting pellets were able to be loaded in mixing chambers via standard hoppers for the production of bituminous conglomerates without significant modification of the production cycle [10]. The fatigue resistance (number of cycles to failure) and the indirect tensile modulus of WTTF-reinforced bituminous were improved by 15% and 55% respectively, compared to cellulose fiber-reinforced samples (Table 4) [10].

**Figure 13:**
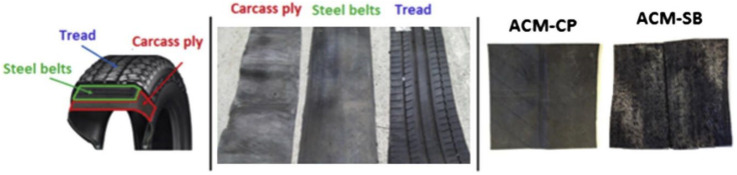
Description of the tire parts used for the development of the ACM systems based on carcass ply (ACM-CP) and steel belts (ACM-SB) [29].

In another work, Navarro et al. [29] used the steel belts (SB) and carcass ply (CP) of ELT (also containing textile fibers) as an anti-reflective cracking mat (ACM) within a pavement structure (Figure 13). It was found that the presence of rubber (elastic particles) in the ACM-CP system delayed the fatigue cracking process by absorbing a portion of the energy applied to the specimen, thus limiting the propagation of fatigue cracks in flexible pavement structures [29].

For fiber-reinforced asphalt, fine rubber particles sticking to tire textile fibers having resilient properties may result in low compaction and increased air voids, making the asphalt mixture susceptible to moisture/water/fluids at high filler content [101]. Bocci et al. [100] evaluated the effect of WTTF for binder layers to increase the mechanical performance of HMA. They claimed that WTTF decreased the HMA mix compatibility, and higher filler content was required to increase the volume of the bituminous mastic up to 5% and to decrease the air voids content to 2.4% (at a compaction energy of 180 gyrations) [100]. They also observed a substantial increase in the HMA resistance to fatigue due to the fibers’ ability to oppose the micro-cracks evolution into macro-cracks by maintaining the micro-crack surfaces closer together [100]. Pais et al. [94] applied fibers as a by-product of used truck and car tires grinding in asphalt mixtures without any treatment. They reported that the tire-derived fiber residues were mainly composed of rubber (about 60%) including fine crumb rubber (33.2%), large crumb rubber (24.2%), string interlaced textile fibers (30.5%), and isolated interlaced textile fibers (12.1%). Although WTTF are susceptible to water absorption affecting the asphalt performance and appearance, the average water absorption of tire textile fibers was only 4.18%, which was half of the value obtained for polyacrylonitrile and polyester fibers [102].

From an environment and economic point of view, improving HMA performances with elevated service life contributes to lower environmental issues of HMA-based pavements with reduced demand for virgin aggregates and bitumen (serving as adhesive to bind the HMA components), road infrastructures and energy consumption [26,27]. In this context, Landi et al. [99] presented a life cycle assessment (LCA) of hot HMA mixtures by evaluating the environmental performance of a standard mixture compared to cellulose-reinforced and WTTF-reinforced HMA mixtures. According to their results, WTTF-reinforced HMA may have longer service lives leading to high amounts of aggregates and bitumen (19.6 kg) being required during a complete life cycle perspective (a period of 30 years was used) compared to a standard HMA (38.3 kg bitumen) and cellulose fiber-reinforced HMA (24.3 kg of bitumen) [99]. Thus, switching from a standard HMA to a WTTF-reinforced HMA may result in a savings of about 405 MJ as a direct result of less bitumen being used and other raw materials being non-renewable petroleum-based resources [99].

To limit global warming and reduce greenhouse gas (GHG) emissions, the asphalt industries made recent efforts to develop new asphalt mixtures called warm-mix asphalt (WMA), which are suitable for lower construction temperatures without the sacrifice of properties [103]. As an example of limited research focusing on WMA reinforced with recycled fibers, Jin et al. [95] investigated the effect of both scrap tire rubber and ELT fiber on WMA modified with Sasobit (synthetic commercial wax). They observed higher crack resistance of tire rubber–fiber-modified WMA by 24.3% and 7.7% compared to conventional HMA and conventional asphalt mixture, respectively [95].

## 5. Rubber Aerogel

Successful application of WTTF in insulation products has attracted a great deal of interest as environmental and economic incentives to recycle waste tire fiber residues [10]. Due to industrialization and rapid urbanization, noise pollution caused by road traffic, construction sites, industries or social activities have raised concerns as important health issues. Prolonged exposure to noisy environments may cause stress, insomnia, cardiac dysfunction and high blood pressure [104]. For instance, the Ministry of Manpower (Singapore) reported in 2019 that noise pollution adversely affected the lives of about 75.49 thousand workers in the industry, having disability or other medical problems [105].

**Figure 14:**
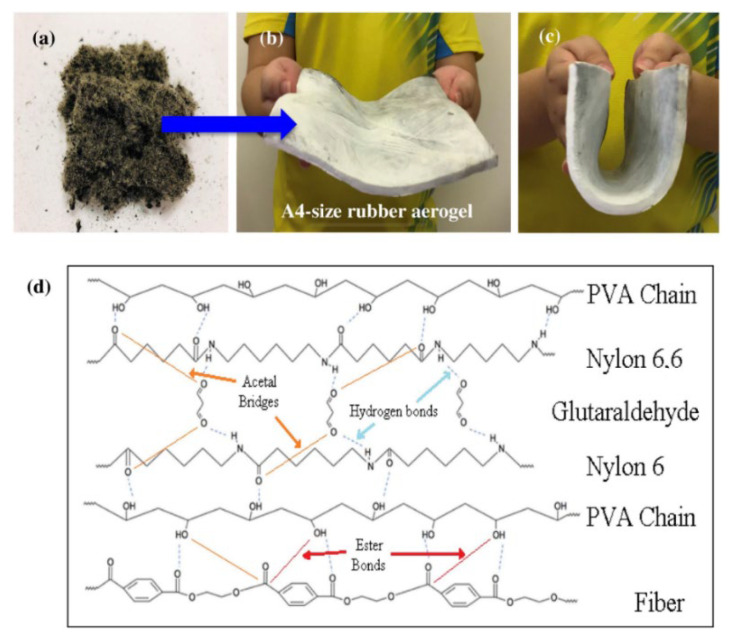
(**a**) Raw WTTF obtained from shredding the car tire waste, (**b**) A4-size rubber aerogel, (**c**) high flexibility of the rubber aerogel and (**d**) proposed formation mechanism between the WTTF and the crosslinkers inside the rubber aerogel [109].

Sound insulators are categorized into resonant and porous insulation materials addressing the above-mentioned issue by absorbing the sound energy during a noise propagation process. Despite the limited efficiency of resonant materials caused by their narrow absorption frequency bands and their heavy weights, porous materials can be applied in a wide range of sound absorption frequencies with a wide range of structural designs [104,106]. However, the high thicknesses of sound absorption materials prevent their efficiency in viscous–thermal acoustic energy practices. This indicates the urgent need to develop high-value products such as aerogels [107]. Aerogels are highly porous (70–99%) materials with an ultralow thermal conductivity (0.012–0.050 W/m.K), an extremely low density (0.003–0.5 g/cm^3^), large specific surface area (520–1590 m^2^/g) and outstanding acoustic insulation properties. These materials are now being used for both noise and thermal insulations/filters (building insulation materials), as well as for oil absorption [107,108]. The development of rubber aerogels having very low density, high porosity and high compressibility not only has wide potential markets (heat and sound insulation, oil cleaning applications, etc.) and environmental benefits (minimize the risks of soil pollution and groundwater contamination by waste tire landfill, reducing the solvent exchange steps in the synthesis procedure by using water instead of toxic organic solvents), but can also address the waste generation issues of the tire industries. Rubber aerogels were first introduced by Thai et al. [109] as they prepared rubber aerogel crosslinkers through a freeze-drying method using WTTF, PVA and glutaraldehyde (GA) (crosslinking agent). The WTTF (30–50 μm diameter, 1–1.8 cm length) were soaked in acetone inducing a hydrolysis reaction, followed by swelling and sonication (80 °C for 20 min) in an acetone/PVA/GA/H_2_O mixture for crosslinking. The cured mixture (85 °C for 3 h) was gelled in a refrigerator and then freeze-dried to obtain a rubber aerogel as shown in Figure 14b. Figure 14d shows that the presence of PVA led to the formation of hydrogen bonds and ester bonds between the carboxyl groups of the polymer chains in WTTF, mainly composed of Nylon 6.6, and the oxide group of PVA chains, while the addition of GA further reinforced the WTTF-PVA structure as a result of acetal bridges being formed between the oxide groups of the polymer fibers and PVA chains [109].

**Figure 15:**
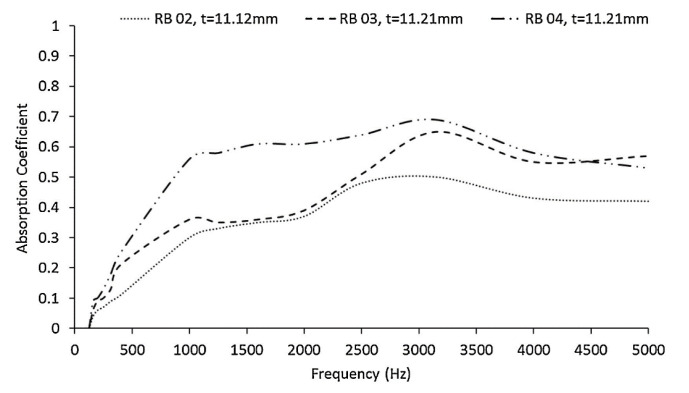
Sound absorption coefficient curves of rubber aerogels at different fiber concentrations (2.0, 3.0, and 4.0 wt.%) [109].

Based on the acoustic insulation properties of rubber aerogels (Figure 15), the 11.2 mm thick aerogel containing 4.0 wt.% WTTF showed the best sound insulation (highest absorption coefficient). This observation can be related to the high fiber–fiber contact area and fiber entanglements at high WTTF loading (4.0 wt.% for RB 04 sample) contributing to more energy being lost (dissipated) by the propagating sound waves due to increasing surface friction and internal frictional losses [109]. The rubber aerogel not only showed better acoustic performance than the commercial foam absorber Basmel^®^, but also its oil absorption capacity was competitive compared to commercial PP and polyurethane (PU) sorbents [109,110,111].

**Figure 16:**
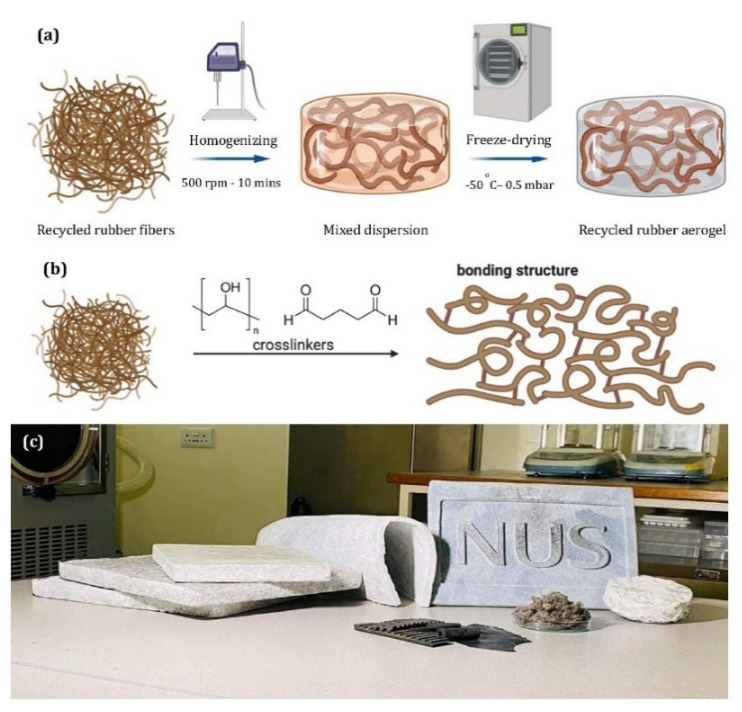
(**a**) Fabrication steps for the recycled rubber aerogels. (**b**) Schematic representation of the bonding structure formation. (**c**) Image presenting typical recycled rubber aerogel samples of different dimensions [114].

From an environmental point of view, it is also of great importance to develop aerogels for the removal of oil contaminants from oil-polluted waterways to safeguard aquatic and terrestrial lifeforms. Although silica aerogels are widely used to cope with oil spills, organic pollutants and industrial oil wastewater, complex and expensive manufacturing combined with the fragility of these aerogels prevent their widespread application, as high mechanical strength is required for specific applications [112,113]. Recent research on rubber aerogels was devoted to design and produce aerogels via a simple method showing very high oil absorption capacity, about two times more than conventional oil sorbents [114]. Figure 16 shows that water, as an eco-friendly solvent, is used for aerogel fabrication, which is very fast (about 15 h) compared to other sol–gel methods [115], taking more than a week to convert the hydrogels into aerogels [114]. The freeze-drying process was used to fabricate rubber aerogels based on WTTF, PVA and co-crosslinkers (GA) without shrinkage. In addition, methyltrimethoxysilane (MTMS) coating was applied onto the aerogel surface, inducing a very stable super-hydrophobic property (water contact angles of 147.9–153.8°) for over a period of six months, contributing to a high absorption capacity of 25.0 g/g. The super-hydrophobicity and high porosity of rubber aerogel promote its oil absorption by providing extra storage space for oil in the inner pores of the aerogel [114]. The addition of fiber up to 1.0 wt.% led to the lowest density (0.020 g/cm^3^) and highest porosity (98.3%) of the aerogels, resulting in a maximum oil absorption capacity (25.0 g/g) similar to cellulose-based aerogels [116], and 2.6 times more important than polyacrylonitrile fiber-reinforced silica aerogels [117]. Furthermore, further increases in fiber content up to 5 wt.% decreased the absorption capacity to only 6.4 g/g due to a more compact network and denser structure, leading to less air pockets and decreasing the porosity and providing less space for oil penetration [114].

**Figure 17:**
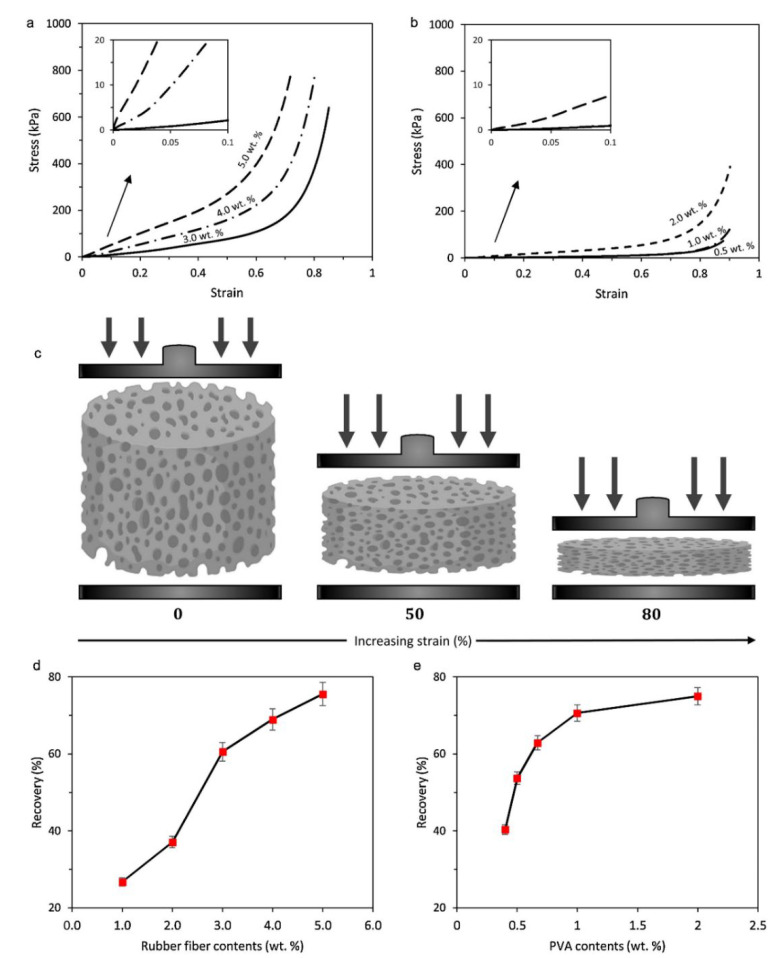
(**a**) Compressive stress–strain curves of rubber aerogels with various contents of WTTF and constant PVA content (1 wt.%). (**b**) Compressive stress–strain curves of rubber aerogels at various PVA contents and constant WTTF content (2 wt.%). (**c**) Schematic description of the changes in the cellular structure with compressive deformation. (**d**,**e**) Recovery performance of rubber aerogels after compression with varying contents of WTTF and PVA [105].

In general, the aerogel fabrication process controls the gel formation, while the drying steps determine the final structure and properties of aerogels [115,118]. Contrary to cellulose-based aerogels or silica aerogels that are highly brittle, the addition of scrap tire textile fibers is expected to improve the mechanical properties of rubber aerogels to undergo high deformation, especially for bending and folding without failure of the porous structure [33,105]. The presence of an interconnected network between the tire fibers and PVA enables the rubber aerogels to withstand the applied stresses and prevents overloading of the fibrous structure, resulting in significant geometric deformation under compression. As shown in compressive strain–stress curves (Figure 17a,b), rubber aerogels present three regions of compressive deformation including elastic deformation (strain ε < 2%), compaction (2% < ε < 60%) and densification (ε > 60%) [105]. The plateau area beyond the yield point is related to the rapidly collapsing porous structure during compressive testing, as the stress slowly increases with increasing compressive strain (ε < 60%), while the specimens experience a sharp increase in stress due to densification at ε > 60% [105,119]. As shown in Figure 17c, deformation of the larger pores (ε > 60%) and densification of the smaller pores beyond 60% strain resulted in a high level of strain absorption. As a function of WTTF content (1–5 wt.%), incorporation of 5 wt.% rubber fiber led to a maximum Young’s modulus of 965.6 kPa, which is much higher than that of Styrofoam (commercial aerogel) and within the range of silica aerogels (0.1–10 MPa) [33,105]. It was observed that increasing the WTTF content increased the compression resistance of aerogels to undergo large deformations without fracture. Figure 17d shows that the introduction of WTTF into the porous structure substantially increased the recovery performance by up to 2.7 times as a result of reduced porosity, thereby decreasing shrinking and preserving the structural integrity of the aerogels [105].

Thermal insulation is an important parameter in the design and fabrication of rubber aerogels, reflecting their overall performance by aiming at energy savings and minimizing carbon emissions. The thermal conductivity of the solid and gas phases play dominant roles on the thermal insulation efficiency of aerogels as lightweight structures. The heat transported via convection in the gas phase due to the displacement of molecules under a pressure gradient should be minimized by partially closed cells and pores smaller than the mean free path of gas (<70 nm) [33,120]. According to the literature, the thermal conductivity of rubber aerogel increases with increasing WTTF content because of more filled up volume at high fiber loading, resulting in lower porosities where the total volume of the voids within the aerogel structures is decreased. Therefore, the thermal conductivity of the rubber aerogels increased due to less air being trapped in the voids [33]. For instance, Thai et al. [109] reported increasing thermal conductivity of rubber aerogels based on WTTF and PVA from 0.035 to 0.047 W/m·K with increasing fiber concentration (1–5 wt.%) as a result of lower porosity (97.1% to 88.0%). Increasing the PVA content from 1 to 2 wt.% at fixed WTTF content (2 wt.%) contributed to lower thermal conductivity (from 0.049 to 0.038 W/m·K) because of increased porosity of the rubber aerogels (93.1% to 95.7%) [109]. The Pham and Phan-Thien [121] model can predict the low thermal conductivity of rubber aerogels indicating that the thermal conductivity of a three-phase composite aerogel (WTTF, PVA, air) is controlled by the volume fraction of each component and the thermal conductivity of air (0.026 W/m·K) [122], WTTF (0.45 W/m·K) [105] and PVA (0.40 W/m·K) [122]. Thus, the low thermal conductivity of rubber aerogels depending on the volume fraction of air can be minimized by varying the tire textile fibers and PVA contents and increasing the overall aerogel porosity [105].

## 6. Blending with Polymers

Polymer composites comprising polymer matrices and fibers (natural/synthetic) are of high interest to achieve good physical, mechanical, thermal and dynamic properties at affordable cost. Fiber-reinforced polymer composites find a wide range of applications in construction, automotive, electronic, packaging and biomedical sectors [123]. Among the different short fibers available, a wide range of natural fibers are now investigated: hemp [124], flax [125], bamboo [126], jute [127], rice husk [128], coconut husk [129], and pineapple leaf [130]. They are extensively being used as reinforcements in composites, especially as alternatives to petroleum-based materials. Natural fibers have significant advantages over synthetic reinforcements (glass, carbon, aramid, etc.) such as low cost, low density, non-toxicity, renewable and biodegradable, with limited waste disposal problems [123]. However, several challenges are associated with natural fibers such as high moisture content/absorption, poor compatibility with most matrices, low fire resistance, and low impact strength [131]. Several reviews are available on the conventional processing routes and critical issues associated with natural fiber-reinforced composites [132], chemical interaction between natural fibers and matrices [133], and effects of interfacial adhesion on the strength of short fiber-reinforced composites [134]. Although a large body of literature is available on hybrid fiber-reinforced composites, there is a limited number of studies on WTTF as reinforcements into polymer composites [43,44,135].

Marconi et al. [136] investigated the environmental effects of reusing tire textile fibers as a second-life material to reinforce plastic compounds. In terms of climate change and fossil depletion, reusing WTTF in plastics compounding is the best scenario to prevent the emissions of toxic substances through fiber incineration and saving fossil fuels (coal, oil, etc.), as well as decreasing the amount of virgin plastics consumed. However, due to a lack of interest for scrap tire textile fibers contaminated with crumb rubber, they are rarely blended with polymer matrices (thermoplastic, thermoset and rubber) [43,44,135]. Additional cleaning processes (centrifugal separation) are required to remove most of the rubber particles (vulcanized) that may act as defects in polymer composites, degrading the performance and durability of the resulting products, as well as making the processing more difficult (extrusion and injection) for a polymer compound [137]. In recent years, WTTF (about 10% by weight of ELT) has gained more interest to develop sustainable polymer composites with potential applications (geotechnical engineering and construction materials, insulation products, polymer compounds, etc.) and to reduce the effect of waste polymers in landfills and toxic gas emission. For example, Zhang et al. [135] used ELT-derived textile short fibers to produce rubber composites from GTR through mechanical milling. They developed a system of stress-induced mechanochemical devulcanization of waste tire rubber mixed with textile fibers through pulverization at ambient temperature. The introduction of 5 wt.% WTTF through co-milling of fibers mixed with GTR resulted in higher tensile strength (9.5 MPa) than pan milling of the compound (6 MPa) due to improved interfacial adhesion minimizing voids formation, possibility at the ends of the fibers during straining, and hence retarding crack initiation [135]. Strong adhesion between the fibers and devulcanized rubber is responsible for better stress transfer from the matrix to the fibers, leading to increased tensile strength and less probability of specimen failure [44]. Rubber compounds with longitudinal fiber direction showed higher tensile properties than in the transverse direction at low fiber content (5 wt.%) due to higher overall strain resistance and growing crack inhibition, while further increases in WTTF content up to 20 wt.% led to fiber entanglement, preventing unidirectional orientation in the composites [135].

**Figure 18:**
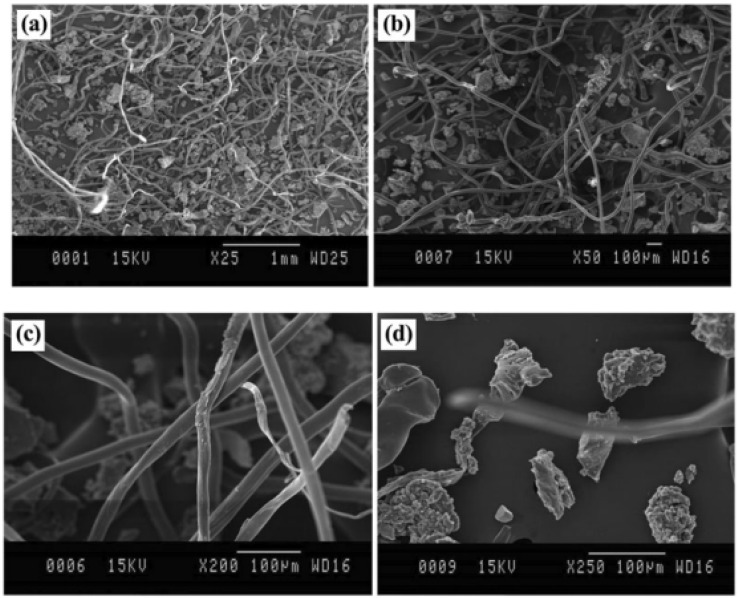
Typical SEM images of WTTF mixed with GTR at different magnifications (**a**–**d**) [43].

Irrespective of fiber cleaning efforts, tire-derived fibers always contain some impurities and rubber crumbs which are major obstacles to their recycling and are used in polymer composites. Nevertheless, Moghaddamzadeh and Rodrigue [43] reported significant amounts of rubber particles sticking on the fluffy fibers with high fiber entanglement. The average diameter of the fibers was about 20 +/− 2 mm (Figure 18c) with an average length of 2500 +/− 1500 mm (Figure 18a,b), while the rubber particles size distribution was roughly 90 +/− 10 mm (Figure 18d). In addition, energy dispersive spectroscopy (EDS) analyses confirmed the presence of impurities such as metal atoms (Al, Cu and Zn) and other additives (processing/vulcanization package) or polymeric materials used in tire formulations [43]. For example, aluminum silicates are common reinforcing fillers in tire structures, while sulfur and zinc oxides are used for rubber curing [138].

**Figure 19:**
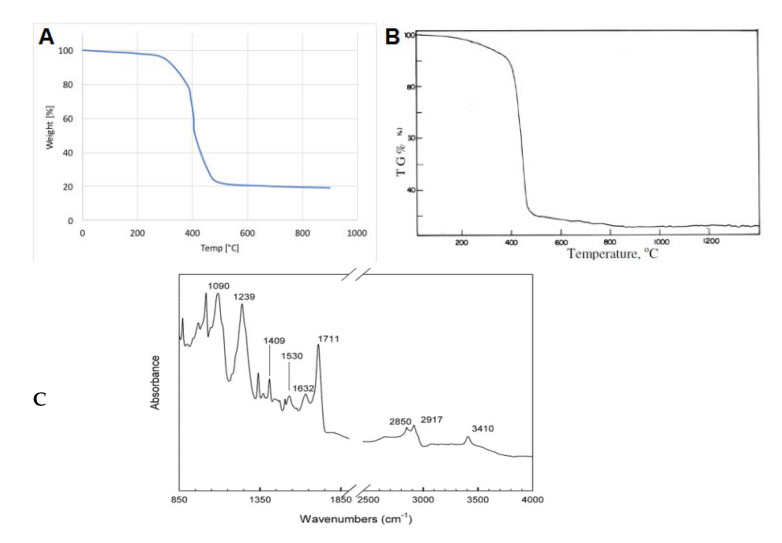
(**A**) TGA of textile materials and (**B**) of Nylon 6.6 [10], as well as (**C**) the FTIR spectrum of WTTF [43].

Landi et al. [10] used TGA on WTTF (Figure 19A) to compare the curve with Nylon 6.6 (Figure 19B) and confirmed the composition of the textile fibers. Furthermore, Moghaddamzadeh and Rodrigue [43] claimed that polyester was the main component of their WTTF using DTG and DSC, as well as FTIR showing the presence of specific peaks for carbonyl (C=O) (1711 cm^−1^), hydroxyl (O–H) (3410 cm^−1^), aromatic ring (1632 and 1409 cm^−1^), aliphatic C–H stretching (2917 and 2850 cm^−1^), and C(O)–O stretching (1239 cm^−1^) of the ester groups in PET (Figure 19C). In a similar work, Onuaguluchi and Banthia used infrared spectra of the as-received WTTF and known polyester fabric to confirm by matching the spectra that both materials were polyester fibers [42]. Thus, the temperature to process WTTF as reinforcement in thermoplastic matrices needs to be lower than the melting point of Nylon 6.6 (259 °C) [46] or PET (253 °C) [43] as the main components of fiber residues. Nevertheless, the thermal degradation of the residual rubber particles on the fiber’s surface is around 180–185 °C [139]. Thus, polyolefins (PP, PE, etc.) having good mechanical properties, excellent processability, low cost and high availability are appropriate matrices for melt blending with WTTF [136].

It is expected that melt blending of a polymer matrix with fiber residues contaminated by rubber particles as vulcanized materials with crosslinked network (not melting) would lead to poor mechanical properties due to low compatibility/interactions between the phases [140]. This is why Ferreira et al. [141] investigated the mechanical recycling of tire textile fibers by using a hybrid system based on recycled polyethylene terephthalate (rPET) reinforced by glass fibers and waste polyamide (W-PA) fibers from scrap tires. They observed low tensile properties of the composites attributed to the presence of the remaining rubber particles in W-PA despite the purification performed in the polyamide fiber wastes.

**Figure 20:**
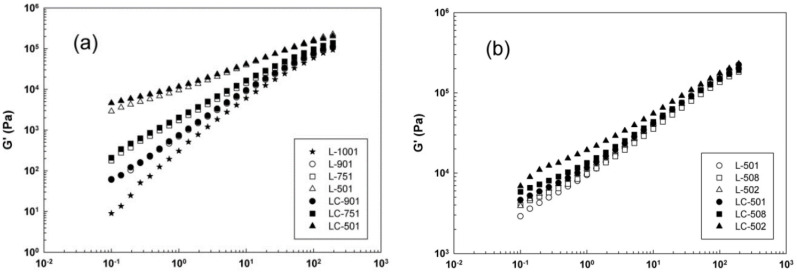
Dynamic elastic modulus as a function of angular frequency to determine the effect of: (**a**) RTF content, and (**b**) extruder screw speed [146].

In agreement with the results of several independent research groups, Fazli and Rodrigue also reported low affinity between thermoplastic polyolefin matrices and GTR particles due to the crosslinked structure of tire rubber (not melting), resulting in poor compatibility and low interfacial adhesion [142,143]. Thus, the tensile strength of recycled high-density polyethylene (rHDPE) decreased from 19.0 to 10.6 MPa with the addition of 20 wt.% GTR, in agreement with similar reports [124,144,145]. Although GTR particles can undergo devulcanization (break-up of the crosslinked network via C-S and/or S-S bonds scission) to promote rubber chain mobility/interaction with the polymer matrix, the rupture of rubber hydrocarbon chains during devulcanization may lead to a drop in molecular weight (MW), thus decreasing the mechanical properties of the resulting composites [2,144]. Therefore, it is of great importance to find a solution for incompatibility issues and performance of tire textile fiber-reinforced composites to compensate for the loss of mechanical properties caused by rubber contamination (stress concentration point). Moghaddamzadeh and Rodrigue used styrene–ethylene–butylene–styrene-grafted maleic anhydride (SEBS-g-MA) as a compatibilizer having a similar structure with polyolefin and fibers to modify the interfacial interactions and mechanical properties of WTTF-reinforced composites [140,146]. Adding 10 wt.% SEBS-g-MA increased the tensile strength of linear low-density polyethylene (LLDPE)/WTTF (75/25) by 14% (10.3 MPa to 11.7 Mpa), which was attributed to improved stress transfer from LLDPE to the reinforcements and good compatibility between styrene (SEBS) and PET (main component of tire textile fibers), ethylene–butylene blocks of the compatibilizer with PE, and maleic anhydride groups with PE, hydroxyl and carboxylic end groups of PET [140]. Figure 20 shows that the introduction of SEBS-g-MA contributed to higher elasticity of the compatibilized samples compared to uncompatibilized ones as a consequence of reduced surface tension between the phases, thus improving interfacial stress transfer from the matrix to fibers. However, the processing conditions also have an important effect on the final properties of multiphase systems. As illustrated in Figure 20b, at constant fiber content (50 wt.%), increasing the extruder screw speed from 110 rpm (L-501) to 180 rpm (L-508) and 250 rpm (L-502) decreased the fiber’s aspect ratio due to increased applied shear, leading to higher surface area and elasticity [146]. Figure 20 also reports on the effect of temperature on the final composites as determined via dynamic rheological measurements, which are very sensitive to the composition and structure on the samples.

According to the literature, incorporation of short fibers into plastics results in significant increases in tensile and flexural modulus (high rigidity) at a cost of reduced elasticity and impact strength, resulting in brittle composites [131,147]. Kakroodi et al. [124] reported that adding 30% hemp fibers to maleic anhydride-grafted polyethylene (MAPE) as the matrix increased the tensile modulus by 67% (from 104 to 184 MPa) and tensile strength by 30% (9.1–11.7 MPa) compared to the neat matrix, which did not break under Charpy impact testing (room temperature). However, the presence of hemp fibers (10–60%) significantly decreased the impact strength (from 369.2 to 127.5 J/m).

**Figure 21:**
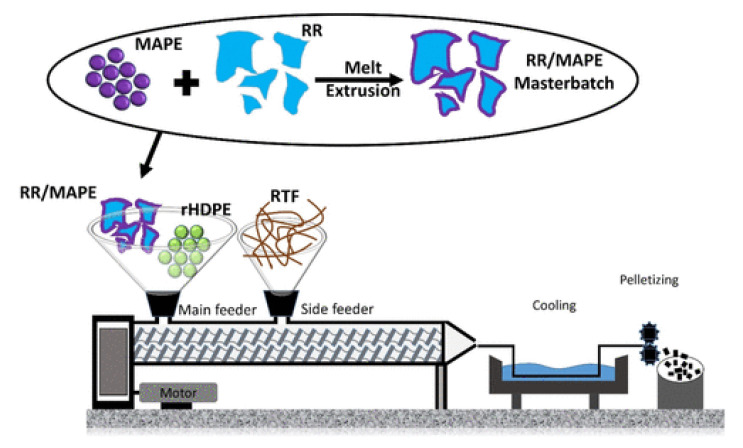
Melt extrusion steps for different rHDPE/(RR/MAPE)/RTF samples [44].

In recent years, the introduction of recycled tire rubber into fiber-reinforced composites is considered as an effective approach for impact strength modification of composites. However, this improvement strongly depends on the blend composition and mixing conditions. Kakroodi and Rodrigue [148] observed higher impact strength of composites (15% flax fiber) by 38% with the addition of only 15% GTR. However, the tensile strength substantially decreased from 17.5 to 11.6 MPa (34% decrease). Fazli and Rodrigue proposed a new approach for impact modification of polymer composites reinforced with RTF through incorporation of a surface-coated RR with compatibilizer [44]. As illustrated in Figure 21, a masterbatch of reclaimed rubber and MAPE was produced prior to melt blending of RTF and the thermoplastic matrix to ensure good surface coverage of rubber particles with the compatibilizer. Table 5 presents the tension and flexion properties of rHDPE-based composites filled with RR, and a mixture of RR/WTTF. The results suggest that melt blending of recycled tire rubber (35–80%) with thermoplastic (rHDPE) led to lower tensile strength (13–4.7 MPa) compared to the matrix (19 MPa) due to very low compatibility between both phases, resulting in voids around rubber particles (stress concentration points) and promoting interfacial debonding. The addition of RTF (20 wt.%) increased the tensile and flexural moduli of fiber-reinforced rubberized composites up to 246.5 and 405.6 MPa, respectively, imparting higher stiffness and resistance to deformation. The interaction of the maleic anhydride group (MAPE) with the hydroxyl group on the carbon black surface or carboxyl groups of rubber particles contributed to improved stress transfer and increased the tensile strength of the compatibilized composites [44,145]. Successful application of the methodology proposed by Fazli and Rodrigue resulted in a substantial increase in impact strength for composites filled with 20% RTF and 45% or 60% of RR/MAPE (70/30) compound leading to 49% (from 246.5 to 368.2 J/m) and 44% (from 275.6 to 398.7 J/m) improvements. The presence of MAPE surface-coated RR delayed crack growth and propagation due to a uniform filler dispersion in the matrix via thick and soft interphase-surrounded rubber, resulting in more energy dissipation [44].

## 7. Conclusions

This review presented a comprehensive overview on the recent progress in waste management of WTTF produced as a waste in ELT treatment. The complexity of the recovery and recycling of WTTF is due to fiber contamination with rubber particles, metal wires and impurities that must be separated during the recycling process to improve the performance of the recycled waste for subsequent reuse as a secondary raw material in future applications. The main conclusions of this review can be classified as:

1. Introduction of WTTF as soil reinforcement contributes to increase the load-bearing capacity of expansive soils while decreasing their swelling. The presence of WTTF in expansive subgrade results in the reduction of permanent deformation under repeated loadings and lower volumetric shrinkage strain.
2. Inclusion of WTTF in concrete composites can minimize plastic shrinkage cracking due to the fibers bridging effect across the cracks, preventing their growth. The fiber bridging effect of WTTF can be enhanced by its hybridization with steel fibers to increase the compressive strength of concrete composites.
3. WTTF have high potential to increase the toughness and fracture resistance of HMA, as well as serving as stabilizers to hinder drain down of the asphalt binder (porous asphalt and SMA mixtures).
4. Tire-derived textile fibers are potential candidates to produce ultralight and highly porous aerogels with strong mechanical performance (to be bent and folded without failure) through simple, cost-effective and time-saving fabrication methods showing great acoustic performance (sound insulation) and oil absorption capacity competitive with commercial sorbents.
5. A fiber cleaning step is highly recommended for melt blending of WTTF with polymers to avoid difficult processing (increasing viscosity) and poor mechanical properties (low compatibility). Recent progress in processing WTTF-reinforced polymer composites reported a substantial increase in impact strength by the introduction of surface-coated fillers to postpone failure due to the presence of a thick and soft interphase surrounding the rubber particles and fibers, resulting in more energy being dissipated before complete failure.

For future developments on WTTF recycling, the next steps should focused on better fiber cleaning efficiency (above 65% fiber purity) using simple processes (mechanical), develop new applications (floor mats, dampers, containers (recycling bins), automotive sector parts, wheels, gaskets, sports equipment, etc.) and find new markets/innovative products made from 100% recycled materials to reduce our environmental impact even more and find the best performance/cost ratio for value-added products.

## Figures and Tables

**Table 1:** Typical tire composition (wt.%) [10].

Type of Tire	Rubber/Elastomers	Carbon Black	Metal	Textile	Zinc Oxide	Others
Passenger Car	47	21.5	16.5	5.5	1	8.5
Lorry	45	22	23	3	2	5
Off Road	47	22	12	10	2	7

**Table 2:** Physical and mechanical characteristics of WTTF [40].

Property	Unit	Testing Method	Values	
			General	Most Frequent
Fiber type	–	–	Yarned	–
Equivalent diameter	mm	ASTM D885M-10A e1 (2014)	0.030–1.50	0.80
Length	mm	ASTM D885M-10A e1 (2014)	0–70	20–40
Tensile strength	MPa	ASTM D885M-10A e1 (2014)	300–2000	600
Twist S (Folded)	T/10 cm	ASTM D885M-10A e1 (2014)	30–50	39
Elongation at break	%	ASTM D885M-10A e1 (2014)	18–25	22
Elastic modulus	GPa	ASTM D885M-10A e1 (2014)	2–7.5	2.7
Hot air shrinkage (at 177 °C × 2 min × 143 g)	%	ASTM D5591-04 (2016)	3–5	4.5
Linear density	Denier	ASTM D885M-10A e1 (2014)	840–1890	1260
Melting point	°C	ASTM D885M-10A e1 (2014)	250–260	256
Water absorption	%	ASTM D885M-10A e1 (2014)	5–13	9.5

**Table 3:** Chemical composition (EDAX result) of WTTF [40].

Element	Symbols	Content (%)
Carbon	C	62.3
Oxygen	O	23.9
Sodium	Na	4.75
Zinc	Zn	2.91
Sulfur	S	2.19
Aluminum	Al	1.83
Silicon	Si	1.38
Magnesium	Mg	0.72

**Table 4:** Comparison between two different types of asphalt with cellulose fibers and ELT fibers [10].

Property	Asphalt with Cellulose Fibers	Asphalt with ELT Fibers
Bitumen (%)	5.4	5.4
Filler (%)	6	6
Indirect tensile module (MPa)	4482	5212
Indirect tensile strength (MPa)	1.37	1.38
Number of cycles to failure	3085	4785

**Table 5:** Mechanical properties of the samples produced [44].

Sample	Tensile Strength (MPa)	Young’s Modulus (MPa)	Tensile Strain at Break (%)	Flexural Modulus (MPa)
rHDPE	19.0 (0.3)	427.1 (14.9)	949.2 (26.4)	594.4 (11.3)
HR35	13.0 (0.3)	191.2 (4.3)	38.1 (4.8)	384.1 (3.5)
HR50	9.2 (0.3)	152.3 (3.2)	44.2 (7.2)	281.8 (5.4)
HR65	7.7 (0.1)	99.3 (4.2)	56.7 (5.3)	189.4 (3.8)
HR80	4.7 (0.4)	32.5 (5.4)	77.9 (8.6)	103.6 (4.7)
HR15F	9.5 (0.1)	246.5 (6.1)	30.2 (6.1)	405.6 (2.1)
HR30F	9.2 (0.3)	170.5 (6.6)	36.4 (4.9)	308.5 (3.8)
HR45F	7.4 (0.2)	109.3 (4.7)	45.3 (6.4)	202.7 (3.5)
HR60F	4.9 (0.1)	45.8 (5.2)	65.2 (5.7)	134.7 (2.9)
HR15F *	13.2 (0.2)	277.3 (4.9)	64.5 (8.2)	437.9 (3.4)
HR30F *	12.1 (0.2)	212.2 (5.3)	87.6 (7.9)	384.6 (4.5)
HR45F *	9.8 (0.1)	126.5 (3.6)	138.2 (7.6)	262.5 (4.2)
HR60F *	8.8 (0.4)	80.9 (4.5)	172.3 (8.3)	182.7 (5.1)
R*x*: RR wt.% (*x*) blended with rHDPE (wt.%). R*x*F: RR wt.% (*x*) blended with 20 wt.% of FF (F) and rHDPE (wt.%). R*y*F *: RR/MPAE wt.% (*y*) blended with 20 wt.% of FF (F) and rHDPE (wt.%).				

## Data Availability

Not applicable.
